# Rearrangement and domestication as drivers of Rosaceae mitogenome plasticity

**DOI:** 10.1186/s12915-022-01383-3

**Published:** 2022-08-19

**Authors:** Manyi Sun, Mingyue Zhang, Xuening Chen, Yueyuan Liu, Binbin Liu, Jiaming Li, Runze Wang, Kejiao Zhao, Jun Wu

**Affiliations:** 1 College of Horticulture, State Key Laboratory of Crop Genetics and Germplasm Enhancement, Nanjing Agricultural University, Nanjing, 210095 Jiangsu China; 2 State Key Laboratory of Crop Biology, College of Horticulture Science and Engineering, Shandong Agricultural University, Tai’an, 271018 China; 3 State Key Laboratory of Systematic and Evolutionary Botany, Institute of Botany, Chinese Academy of Sciences, Beijing, 100093 China; 4 Department of Botany, National Museum of Natural History, Smithsonian Institution, PO Box 37012, Washington, DC, 20013-7012 USA

**Keywords:** Mitogenome, Rosaceae, Rearrangement rate, Domestication

## Abstract

**Background:**

The mitochondrion is an important cellular component in plants and that functions in producing vital energy for the cell. However, the evolution and structure of mitochondrial genomes (mitogenomes) remain unclear in the Rosaceae family. In this study, we assembled 34 Rosaceae mitogenomes and characterized genome variation, rearrangement rate, and selection signal variation within these mitogenomes.

**Results:**

Comparative analysis of six genera from the Amygdaloideae and five from the Rosoideae subfamilies of Rosaceae revealed that three protein-coding genes were absent from the mitogenomes of five Rosoideae genera. Positive correlations between genome size and repeat content were identified in 38 Rosaceae mitogenomes. Twenty repeats with high recombination frequency (> 50%) provided evidence for predominant substoichiometric conformation of the mitogenomes. Variations in rearrangement rates were identified between eleven genera, and within the *Pyrus*, *Malus*, *Prunus*, and *Fragaria* genera. Based on population data, phylogenetic inferences from *Pyrus* mitogenomes supported two distinct maternal lineages of Asian cultivated pears. A *Pyrus*-specific deletion (DEL-D) in selective sweeps was identified based on the assembled genomes and population data. After the DEL-D sequence fragments originally arose, they may have experienced a subsequent doubling event via homologous recombination and sequence transfer in the Amygdaloideae; afterwards, this variant sequence may have significantly expanded to cultivated groups, thereby improving adaptation during the domestication process.

**Conclusions:**

This study characterizes the variations in gene content, genome size, rearrangement rate, and the impact of domestication in Rosaceae mitogenomes and provides insights into their structural variation patterns and phylogenetic relationships.

**Supplementary Information:**

The online version contains supplementary material available at 10.1186/s12915-022-01383-3.

## Background

As the cell’s energy factory, the mitochondrion is an organelle essential in angiosperm development, growth, programmed cell death, and male sterility [1]. Each mitochondrion has its own genome, which is usually uniparentally inherited [2]. Compared with plastid genomes, angiosperm mitogenomes vary in size and gene content [3, 4]. Currently known angiosperm mitochondrial genome (mitogenome) sizes range from 66 kb to 11.3 Mb, and the number of protein-coding genes ranges from 19 to 41 (excluding duplicated genes and open reading frames (ORFs)) [4–6]. Most genome size and structure variations occur in non-coding sequences, and these variations are primarily caused by foreign sequence importation, which increases the occurrence of repetitive sequences and recombination events [7–9].

Numerous inverted and direct repeats play a pivotal role in plant mitogenome size and structural evolution by participating in genome rearrangement, repeat-mediated recombination, insertion, and deletion events [8, 10, 11]. Repeat-mediated homologous recombination in mitogenomes has been investigated in angiosperm plants such as *Picea abies* [12] and *Nymphaea colorata* [13], and positive correlations between repeat length and recombination rate were detected in *Viscum scurruloideum* [4]. Minor to moderate recombination activity was detected among short (< 100 bp) and medium length repeats (100–1000 bp), while larger repeats (> 1000 bp) experienced more frequent recombination activity and isomerization in the genome [14, 15]. Recently, third-generation long-read sequencing technologies have been used to overcome the complexity of short read-based genome assembly, and this technology has proven sensitive at detecting the repeat-mediated recombination activity of large repeats [12, 13].

Mitogenome rearrangement is primarily caused by frequent repeat-mediated recombination [11], supported by the presence of rearrangement breakpoints close to repeats [8]. In plants, mitogenome rearrangements can influence ATP availability, plant growth, cytoplasmic male sterility (CMS), and overall fitness [16, 17]. Aside from the low substitution rate, mitogenomes in angiosperms have obviously different rearrangement rates. Within the genus *Monsonia*, the mitogenome of *M. ciliata* has a tenfold higher rearrangement rate than its sister species; overall, an over 600-fold variance in mitogenome rearrangement rates has been observed among seed plants [8].

Rosaceae has ca. 3000 species in 90 genera and includes herbs, shrubs, and trees adapted to a wide variety of environments [18]. Research on Rosaceae mitogenomes has remained limited despite recent progress in nuclear and chloroplast genomic sequencing of Rosaceae species [19–21]. Only 14 Rosaceae mitogenomes were available in the National Center for Biotechnology Information (NCBI) database (last access date: 20 January 2022) (Additional file 1). The evolution and divergence of Rosaceae mitogenomes remain unclear, and limited genetic information regarding Rosaceae mitogenome analysis exists. Third-generation long-read sequencing technologies and a series of assembly software like GetOrganelle [22], SOAPdenovo [23], and Canu [24] have provided the ability to assemble complete mitogenomes. In addition, many Rosaceae nuclear genomes can only employ different parental inheritance modes, such as in the *Malus*, *Pyrus*, and *Sorbus* genera; information on mitogenomes inherited from the maternal parent provides a chance to determine additional population and domestication information [25, 26].

Here, 38 complete Rosaceae mitogenomes were assembled and annotated (four of which were previously released). Variations in genes and repeat sequences were identified, and recombination and rearrangement events were investigated to explore the expansion and evolution patterns of Rosaceae mitogenomes. Subsequently, short-read sequencing data from 139 pear and 116 apple accessions was used to explore the genetic variations, phylogenetic relationships, and domestication processes in the *Pyrus* and *Malus* genera. We found that domestication and selection contributed to the variations in the mitogenomes of members of the Rosaceae family and resulted in the spread of structurally varied gene sequences.

## Results

### Profile of the mitogenomes of Rosaceae species

**Fig. 1:**
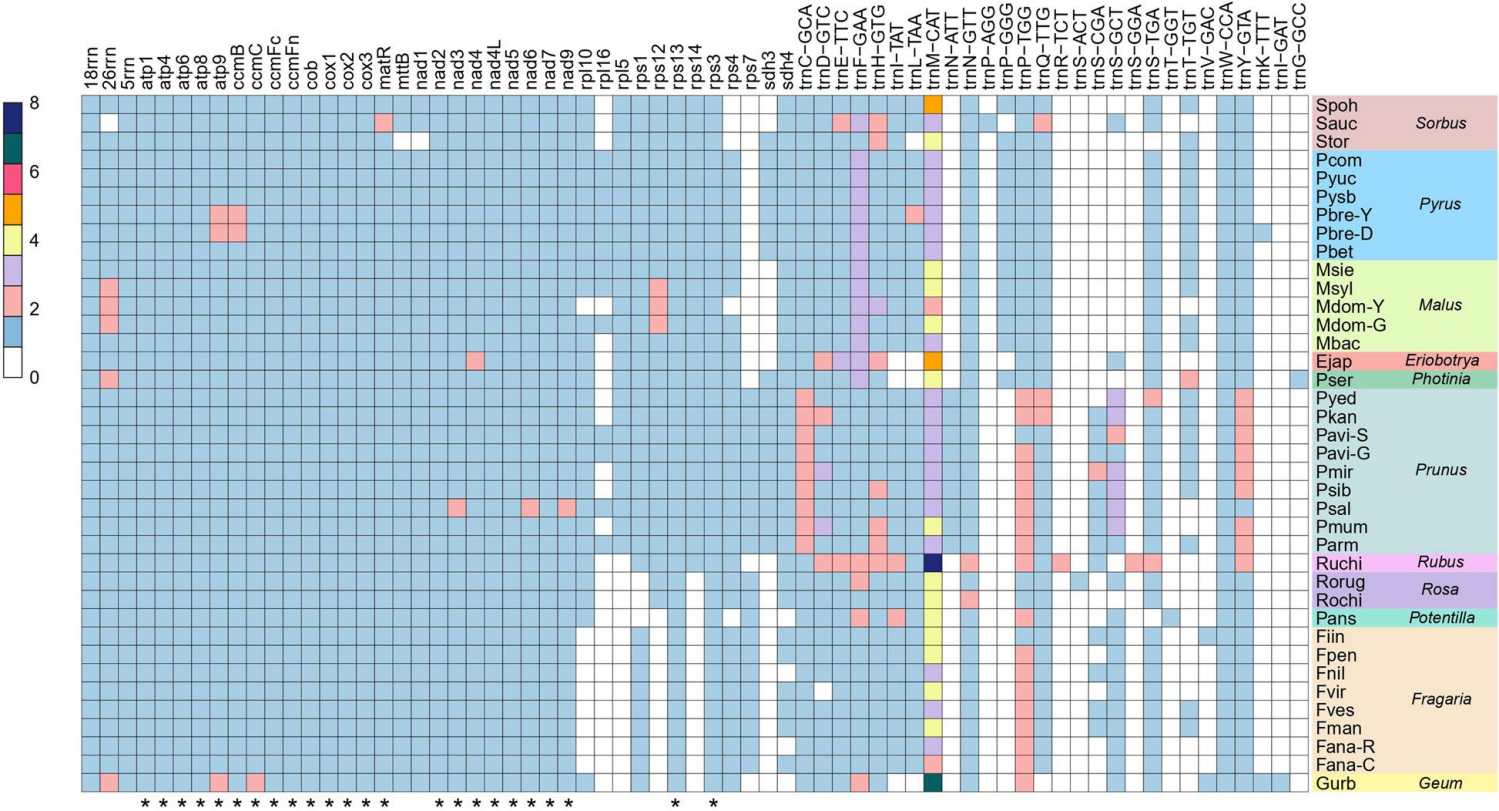
Gene content of 38 mitogenomes. Different colors represent the number of genes present in each mitogenome: Fiin, *Fragaria iinumae*; Fpen, *Fragaria pentaphylla*; Fvir, *Fragaria viridis*; Fman, *Fragaria mandschurica*; Fnil, *Fragaria nilgerrensis*; Fves, *Fragaria vesca*; Fana-C, *Fragaria ananassa* cv. “Camarosa”; Fana-R, *Fragaria ananassa* cv. “Royal Royce”; Rochi: *Rosa chinensis*; Rorug: *Rosa rugosa*; Pans, *Potentilla anserina*; Ruchi, *Rubus chingii*; Mbac, *Malus baccata*; Mdom-G, *Malus domestica* cv. “Gala”; Mdom-Y, *Malus domestica* cv. “Yantai fuji 8”; Msie, *Malus sieversii*; Msyl, *Malus sylvestris*; Pyed, *Prunus yedoensis*; Pkan, *Prunus kanzakura*; Pmir, *Prunus mira*; Pmum, *Prunus mume*; Psib, *Prunus sibirica*; Pavi-G, *Prunus avium* cv. “Glory”; Pavi-S, *P. avium* cv. “Staccato”; Parm, *Prunus armeniaca*; Psal, *Prunus salicina*; Pbet, *Pyrus betulifolia*; Pbre-D, *Pyrus bretschneideri* cv. “Dangshansuli”; Pbre-Y, *Pyrus bretschneideri* cv. “Yali”; Pcom, *Pyrus communis*; Pysb, *Pyrus sinkiangensis* × *bretschneideri*; Pyuc, *Pyrus ussuriensis* × *communis*; Pser, *Photinia serratifolia*; Gurb, *Geum urbanum*; Stor, *Sorbus torminalis*; Spoh, *Sorbus pohuashanensis*; Sauc, *Sorbus aucuparia*; Ejap, *Eriobotrya japonica*. “*” at the bottom of the heatmap means these protein-coding genes appeared in all 38 mitogenomes. The right color bar represents the genus of each of the 38 mitogenomes

In this study, each of the 34 Rosaceae mitogenomes was de novo assembled into a single completely gapless contig with an average coverage depth of 323.52–6550.87× (Additional file 2). Coupled with the four previously released genomes, the sizes of the 38 Rosaceae mitogenomes ranged from 277.76 kb (*Rosa chinensis*: Rochi) to 535.73 kb (*Prunus mume*: Pmum) (Table 1). Genome sizes within the Amygdaloideae varied by up to 150.75 kb (*Sorbus aucuparia*: Sauc (384.98 kb) vs Pmum (535.73 kb)), and this variation increased up to 194.38 kb in Rosoideae species (Table 1). Twenty-four genes appeared in all 38 mitogenomes. Compared with six genera in Amygdaloideae, three protein-coding genes (*rpl5*, *rpl16*, and *sdh3*) were completely lost from the mitogenomes of five genera of the Rosoideae (Fig. 1). Within Rosoideae, *rps14* was lost in *Rosa*, *Geum*, *Potentilla*, and *Fragaria*, and *rps12* was lost in *Geum*, *Potentilla*, and *Fragaria*. *Rpl10* was lost in *Fragaria*, and *rps1* was lost in *Rosa* and *Potentilla*. In six Amygdaloideae genera, *rps7* was lost in *Sorbus*, *Photinia*, *Malus*, *Eriobotrya*, and *Pyrus*. Two varieties of *Pyrus bretschneideri* (“Yali”: Pbre-Y and “Dangshansuli” Pbre-D) contained copies of the *atp9* and *ccmB* genes. *Malus sylvestris* (Msyl) and *Malus domestica* (“Gala”: Mdom-G; “Yantai fuji 8”: Mdom-Y) contained copies of the *26rrn* and *rps12* genes. The GC content was relatively stable, averaging at about 45% in the 38 Rosaceae mitogenomes, except for the *Rubus chingii* (Ruchi) mitogenome (43.31%), which had a higher percent of chloroplast sequence imports relative to other species (Table 1).

**Table 1:** Summary of 38 Rosaceae mitogenomes

ID	Species name	Subfamily	GenBank accession number	Genome size (bp)	Number of protein-coding genes	Number of rRNA	Number of tRNA	GC content (%)	Length of plastid-derived sequences (bp/%)
Ejap	*Eriobotrya japonica*	Amygdaloideae	NC_045228	434,980	34	3	23	45.42	2039 (0.47)
Fana-C	*Fragaria ananassa* (“Camarosa”)^a^	Rosoideae	OM763767	285,543	30	3	17	44.96	19,176 (6.72)
Fana-R	*Fragaria ananassa* (“Royal Royce”)	Rosoideae	OM763768	285,046	29	3	18	44.98	18,507 (6.49)
Fiin	*Fragaria iinumae*	Rosoideae	OM763769	329,263	30	3	20	45.26	26,297 (7.99)
Fman	*Fragaria mandschurica*	Rosoideae	ON478154	313,022	30	3	21	45.20	14,598 (4.66)
Fnil	*Fragaria nilgerrensis*	Rosoideae	OM763770	315,210	29	3	19	45.26	11,742 (3.73)
Fpen	*Fragaria pentaphylla*	Rosoideae	ON478155	290,970	30	3	19	45.07	12,360 (4.25)
Fves	*Fragaria vesca*	Rosoideae	ON478179	312,993	30	3	20	45.20	14,340 (4.58)
Fvir	*Fragaria viridis*	Rosoideae	ON478156	289,146	30	3	18	45.23	12,354 (4.27)
Gurb	*Geum urbanum*	Rosoideae	ON556624	335,549	31	4	23	44.42	30,222 (9.01)
Mbac	*Malus baccata*	Amygdaloideae	ON478159	400,769	33	3	20	45.40	4049 (1.01)
Mdom-G	*Malus domestica* (“Gala”)	Amygdaloideae	ON478160	396,946	35	4	21	45.41	2187 (0.55)
Mdom-Y	*Malus domestica* (“Yantai fuji 8”)	Amygdaloideae	MN964891	396,947	32	4	20	45.40	1968 (0.50)
Msie	*Malus sieversii*	Amygdaloideae	ON478161	385,869	34	3	21	45.38	2140 (0.55)
Msyl	*Malus sylvestris*	Amygdaloideae	ON478162	396,940	35	4	21	45.41	1968 (0.50)
Pans	*Potentilla anserina*	Rosoideae	ON478170	294,682	28	3	21	44.46	14,320 (4.86)
Parm	*Prunus armeniaca*	Amygdaloideae	ON478164	510,346	36	3	24	45.43	5483 (1.07)
Pavi-G	*Prunus avium* (“Glory”)	Amygdaloideae	ON478157	444,576	36	3	23	45.62	3887 (0.87)
Pavi-S	*Prunus avium* (“Staccato”)	Amygdaloideae	ON478178	444,576	36	3	23	45.62	3887 (0.87)
Pbet	*Pyrus betulifolia*	Amygdaloideae	ON478165	432,493	35	3	20	45.21	2039 (0.47)
Pbre-D	*Pyrus bretschneideri* (“Dangshansuli”)	Amygdaloideae	OM763766	458,897	37	3	21	45.21	3901 (0.85)
Pbre-Y	*Pyrus bretschneideri* (“Yali”)	Amygdaloideae	ON478180	458,895	37	3	21	45.21	3900 (0.85)
Pcom	*Pyrus communis*	Amygdaloideae	ON478166	443,525	35	3	20	45.24	4195 (0.95)
Pkan	*Prunus kanzakura*	Amygdaloideae	ON478167	422,215	35	3	27	45.54	3975 (0.94)
Pmir	*Prunus mira*	Amygdaloideae	ON478168	429,732	35	3	28	45.59	2320 (0.54)
Pmum	*Prunus mume*	Amygdaloideae	ON478169	535,727	35	3	28	45.45	5768 (1.08)
Psal	*Prunus salicina*	Amygdaloideae	ON478171	508,005	39	3	23	45.43	3057 (0.60)
Pser	*Photinia serratifolia*	Amygdaloideae	ON556623	473,561	34	4	21	45.25	2062 (0.44)
Psib	*Prunus sibirica*	Amygdaloideae	ON478172	510,187	35	3	26	45.42	5905 (1.16)
Pyed	*Prunus yedoensis*	Amygdaloideae	ON478173	456,900	35	3	26	45.56	3883 (0.85)
Pysb	*Pyrus sinkiangensis x bretschneideri* (“Hongxiangsu”)	Amygdaloideae	ON478158	441,852	35	3	20	45.21	2039 (0.46)
Pyuc	*Pyrus ussuriensis x communis* (“No.1 Zhongai”)	Amygdaloideae	ON478163	441,853	35	3	20	45.21	2039 (0.46)
Rochi	*Rosa chinensis*	Rosoideae	ON478174	277,763	31	3	18	45.35	6000 (2.16)
Rorug	*Rosa rugosa*	Rosoideae	ON478175	302,947	31	3	20	45.24	8863 (2.93)
Ruchi	*Rubus chingii*	Rosoideae	ON478176	472,138	32	3	35	43.31	77,163 (16.34)
Sauc	*Sorbus aucuparia*	Amygdaloideae	NC_052880	384,977	33	2	23	45.39	3763 (0.98)
Spoh	*Sorbus pohuashanensis*	Amygdaloideae	ON478177	396,857	32	3	20	45.36	3044 (0.77)
Stor	*Sorbus torminalis*	Amygdaloideae	NC_052879	386,758	31	3	18	45.31	3837 (0.99)

^a^The accession name is shown in parentheses

### Repeat sequence variation and correlations between genome size and repeat sequences

**Fig. 2:**
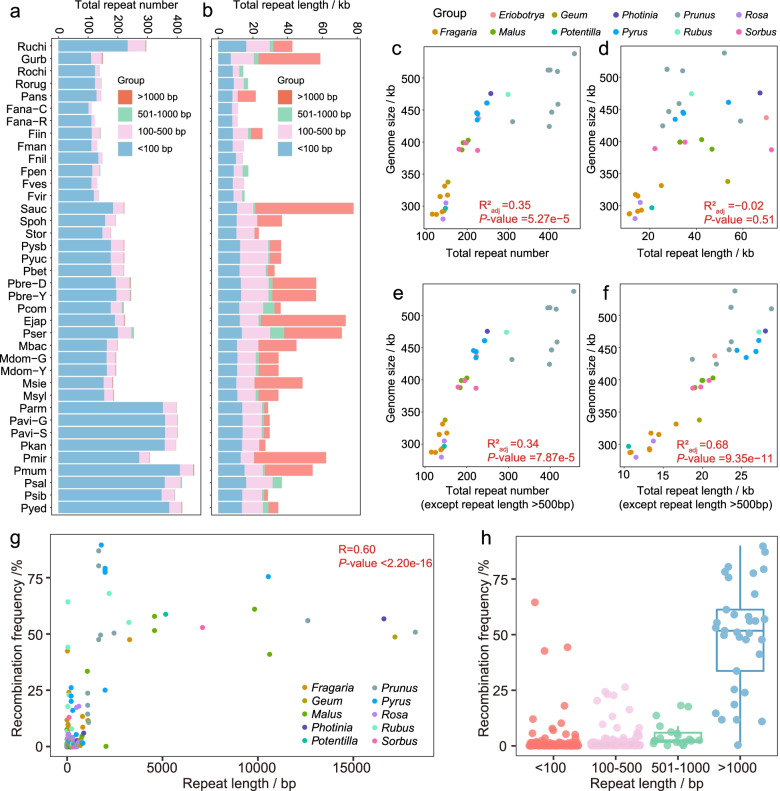
Repeat content and repeat-mediated homologous recombination among Rosaceae mitogenomes. Number of total repeats (**a**) and total repeat length (**b**) of 38 Rosaceae mitogenomes. Red indicates repeat lengths were > 1000 bp; green indicates repeat lengths ranged from 501 to 1000 bp; pink indicates repeat lengths ranged from 100 to 500 bp; blue indicates repeat lengths were < 100 bp. **c**–**f** The correlation between genome size and total repeat number (**c**), total repeat length (**d**), total repeat number (repeat length ≤ 500 bp) (**e**), and total repeat length (repeat length ≤ 500 bp) (**f**) in 38 Rosaceae mitogenomes. *R*^2^ indicates the coefficient of determination, and the *P*-value was determined by *F*-statistic. **g** Repeat-mediated recombination in 33 mitogenomes of ten genera. Each point represents a pair of repeats, and the *y*-axis represents the proportion of mapping to expected recombination products. *R* indicates the correlation coefficient, and the *P*-value was determined by a two-tailed Student’s *t*-test. **h** Distribution of recombination frequencies of the four repeat types based on repeat length (< 100 bp, 100 bp ≤ repeat length ≤ 500 bp, 501 bp ≤ repeat length ≤ 1000 bp, and repeat length > 1000 bp)

In the 38 mitogenomes, total repeat number changes might be caused by short (< 100 bp) repeat sequences (Fig. 2a; Additional file 3). The number of repeat sequences ranged from 112 (*Fragaria ananassa* cv. “Camarosa”: Fana-C) to 457 (Pmum), and 73.33–90.98% of repeats were less than 100 bp in length (Fig. 2a; Additional file 3). Among species of the Amygdaloideae, the number of short (< 100 bp) repeats in *Prunus* samples was significantly higher than samples from five other genera (*Photinia*, *Malus*, *Pyrus*, *Sorbus*, and *Prunus*) (*t*-test, *P*-value = 5.64e−8), while the number of repeats longer than 100 bp was not significantly increased (*t*-test, *P*-value = 0.11) (Additional file 3). In Rosoideae, the total repeat number (296) of Ruchi was higher than that of samples from four genera (total repeat number: 112–150) (Fig. 2a), but the total repeat length in Ruchi was lower than in *Geum urbanum* (Gurb) (Fig. 2b).

For all of the 38 Rosaceae samples, genome size showed a significantly high correlation with repeat number (phylogenetic generalized least squares: PGLS, *R*^2^_adj_ = 0.35, *P*-value = 5.27e−5) (Fig. 2c). In addition, mitogenome size showed significantly high (*P*-value < 0.01) correlations with total repeat number and length (repeat length ≤ 500 bp) (Fig. 2e, f; Additional file 4: Fig. S1 a, b, e, f). However, negligible correlations (*R*^2^_adj_ = − 0.02, *P*-value = 0.51) appeared between total repeat length and genome size (Fig. 2d), and repeats longer than 1000 bp also showed low correlation with genome size (*R*^2^_adj_ = − 0.02 and − 0.03) (Additional file 4: Fig. S1d, h). In 14 Fabaceae mitogenomes, genome size also showed high correlation with repeat sequences (total repeat number: *R*^2^_adj_ = 0.73, *P*-value < 1.00e−3; total repeat length: *R*^2^_adj_ = 0.68, *P*-value < 1.00e−3) (Additional file 4: Fig. S2a, g). All three repeat categories (length < 100 bp, 100 bp ≤ repeat length ≤ 500 bp, and length ≤ 500 bp) showed significant correlation with variations in genome size (*R*^2^_adj_ ranged from 0.67 to 0.90, *P*-value < 1.00e−3) (Additional file 4: Fig. S2b, c, f, h, i, l).

However, in regard to the total number of repeats, repeats shorter than 100 bp or 500 bp showed negligible correlations (*R*^2^_adj_ = 0.10, 0.08, and 0.11) with genome size in 88 (one sample per species) seed plant mitogenomes (Additional file 4: Fig. S3a-c), and repeats > 500 bp showed high correlations with genome size (Additional file 4: Fig. S3d, e). Total repeat length (except for length > 1000 bp) showed significant correlation (*R*^2^_adj_ ranged from 0.45 to 0.51, *P*-value < 1.00e−3) with genome size (Additional file 4: Fig. S3g-j, l). In addition, several mitogenomes may have an overrepresentation of repeat content (Additional file 5). For example, *Hyoscyamus niger* (total repeat length: 133.17 kb) had a similar genome size to *Prunus salicina* (Psal) (501.40 vs. 508.00 kb), but the total repeat length was about 3.97-fold of Psal (33.56 kb). Additionally, 50 mitogenomes, with genome sizes ranging from 271.60 to 525.67 kb, were selected, and 38.49- and 80.47-fold changes in total repeat length and numbers of repeat sequences were identified (Additional file 4: Fig. S4; Additional file 5).

### Recombination of repetitive sequences

Based on long sequencing reads of 33 samples in ten genera, repeat length showed a relatively high correlation with recombination frequency (Pearson correlation coefficient: *R* = 0.60, *P*-value < 2.2e−16) (Fig. 2g). Higher recombination frequencies were observed for long (> 1000 bp) repeats than for medium (100–500 bp and 501–1000 bp) or short (< 100 bp) repeats (Fig. 2h), and percentages of long repeats associated with homologous recombination were higher than that of short and medium repeats (Table 2). A total of 341 recombination events were identified, and 1–35 recombination events appeared in each of the 33 mitogenomes (Additional file 6). Among short repeats (< 100 bp), only 2.45% (164/6707), underwent homologous recombination, but this percentage increased to 86.84% (33/38) for the long repeats (> 1000 bp) (Table 2). Among the 341 repeats exhibiting recombination activity, 81.82% (27/33) of the long repeats recombined with a frequency greater than 20%, and 83.54% (137/164) of the short repeats had recombination frequencies lower than 1%. Twenty repeats had over 50% recombination frequency (Additional file 6). In *Pyrus*, a repeat of 2040 bp length and 25.31% recombination frequency in Pbre-Y exhibited 77.61–79.37% recombination frequency in “Hongxiangsuli” (*Pyrus sinkiangensis* × *bretschneideri*: Pysb), *Pyrus betulifolia* (Pbet), and “No.1 Zhong’ai” (*Pyrus ussuriensis* × *communis*: Pyuc). In *Pyrus communis* (Pcom), this repeat was shortened to 1841 bp, and the recombination frequency reached 89.69% (Additional file 6).

**Table 2:** Recombination statistics on four types of repeats among 33 mitogenomes

Sample	Number (%^a^) of repeats^b^ with recombination activity			
	Repeat length < 100 bp	100 bp ≤ repeat length ≤ 500 bp	500 bp < repeat length ≤ 1000 bp	Repeat length > 1000 bp
Fana-C	2 (1.98)	1 (9.09)	NA	NA
Fana-R	11 (9.91)	6 (54.55)	NA	NA
Fiin	3 (2.68)	1 (3.70)	0 (0.00)	1 (100.00)
Fman	3 (2.70)	1 (5.00)	NA	NA
Fnil	8 (5.97)	1 (6.67)	NA	NA
Fpen	2 (1.75)	3 (12.50)	3 (100.00)	NA
Fves	0 (0.00)	1 (5.00)	NA	NA
Fvir	2 (1.68)	1 (6.25)	1 (100.00)	NA
Mbac	6 (3.68)	11 (32.35)	NA	1 (50.00)
Mdom-G	4 (2.47)	3 (10.34)	1 (100.00)	2 (100.00)
Msie	1 (0.66)	1 (3.45)	NA	2 (66.67)
Msyl	2 (1.30)	5 (16.67)	1 (100.00)	1 (50.00)
Pans	1 (0.78)	2 (13.33)	NA	1 (100.00)
Parm	7 (1.99)	7 (15.91)	0 (0.00)	1 (100.00)
Pavi-G	4 (1.11)	0 (0.00)	0 (0.00)	1 (100.00)
Pavi-S	4 (1.11)	1 (2.44)	0 (0.00)	1 (100.00)
Pbet	5 (2.81)	5 (12.20)	0 (0.00)	1 (100.00)
Pbre-Y	5 (2.56)	9 (20.00)	1 (33.33)	2 (100.00)
Pcom	2 (1.14)	0 (0.00)	1 (16.67)	1 (100.00)
Pkan	7 (1.96)	0 (0.00)	NA	1 (100.00)
Pmir	11 (4.04)	6 (18.18)	NA	2 (100.00)
Pmum	6 (1.47)	3 (6.82)	0 (0.00)	3 (100.00)
Psal	0 (0.00)	0 (0.00)	3 (60.00)	NA
Psib	4 (1.15)	0 (0.00)	0 (0.00)	1 (100.00)
Pyed	13 (3.49)	19 (47.50)	1 (50.00)	2 (100.00)
Pysb	3 (1.69)	6 (14.29)	0 (0.00)	1 (50.00)
Pyuc	3 (1.70)	2 (4.65)	1 (100.00)	1 (50.00)
Rochi	21 (17.07)	6 (46.15)	2 (100.00)	NA
Rorug	3 (2.44)	0 (0.00)	2 (100.00)	NA
Ruchi	6 (2.58)	6 (10.17)	0 (0.00)	2 (100.00)
Spoh	5 (3.18)	10 (28.57)	NA	1 (100.00)
Gurb	7 (6.36)	4 (11.43)	0 (0.00)	3 (100.00)
Pser	3 (1.50)	4 (8.70)	2 (28.57)	1 (100.00)
Total	164 (2.45)	125 (11.64)	19 (40.43)	33 (86.84)

“NA” means repeat type was in the sample

^a^Percent = number of repeats with recombination/number of total repeats × 100%

^b^Repeats were divided into four types including short repeats (length < 100 bp), two types of medium repeats (100 bp ≤ length ≤ 500 bp and 500 bp < length ≤ 1000 bp), and long repeats (length > 1000 bp)

### Rearrangement rates of the 38 mitogenomes

**Fig. 3:**
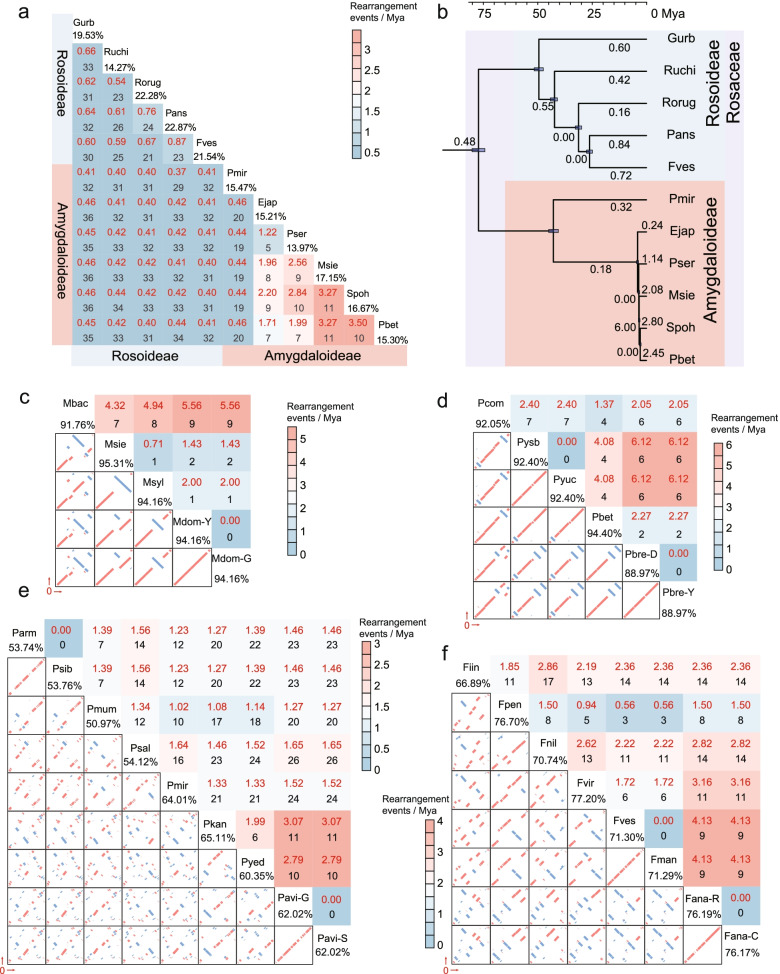
Rearrangement event and rate analysis. **a** Number and rate of pair-wise rearrangement events between eleven genera. Black numbers represent the rearrangement events (pair-wise rearrangement events), and red numbers represent the pair-wise rearrangement rates. **b** Rearrangement rates in eleven genera. The numbers of rearrangement events per million years are displayed on branches of the phylogeny. **c**–**f** The pair-wise analysis of rearrangement events and rate within *Malus* (**c**), *Pyrus* (**d**), *Prunus* (**e**), and *Fragaria* (**f**). Upper-right heatmaps represent the pair-wise rearrangement rates. Red numbers represent the rearrangement rates, and black numbers represent the rearrangement events. Bottom-left figures display the synteny analysis between two samples. Red represents direct, and blue represents inverted. Numbers under the sample ID represent the percent of shared sequences

Repeat-mediated recombination may further contribute to the rearrangement of mitogenomes [8]. In this study, eleven mitogenomes (*Prunus mira*: Pmir, *Fragaria vesca*: Fves, *Eriobotrya japonica*: Ejap, Pbet, Ruchi, *Rosa rugosa*: Rorug, *Potentilla anserina*: Pans, *Photinia serratifolia*: Pser, *Geum urbanum*: Gurb, *Sorbus pohuashanensis*: Spoh and *Malus sieversii*: Msie) were chosen to represent eleven genera. About 13.97–22.87% of the mitochondrial sequences were shared among all eleven genera, and more than 29 rearrangements were identified between the Amygdaloideae and Rosoideae subfamilies (Fig. 3a). Twenty-one to 33 rearrangement events were identified in five Rosoideae genera, and 5–20 rearrangement events were identified in six Amygdaloideae genera. The rearrangements were then evaluated within each genus to avoid complications resulting from the high sequence divergence between genera [12]. In *Malus*, 91.76–95.31% of sequences were shared (Fig. 3c), and seven to nine rearrangement events were identified between *Malus baccata* (Mabc) and the other four apples. One or two rearrangements were detected between the remaining four accessions (Msie, Msyl, Mdom-Y, and Mdom-G). About 88.97–94.40% of sequences appeared within the six pears (Fig. 3d), and four to seven rearrangements were identified between Pcom and the other five Asian pears. Among the four Asian cultivated pears studied, six rearrangement events were identified between the Pysb and Pyuc and the Pbre-D and Pbre-Y species. Unexpectedly, no rearrangement events were detected between Pysb and Pyuc, despite their maternal parents coming from two pear systems (*Pyrus sinkiangensis* and *Pyrus ussuriensis*) (Fig. 3d). In *Prunus*, only 50.97–65.11% of sequences were shared, and 0–26 rearrangements were identified (Fig. 3e). In *Fragaria*, 66.89–77.20% homologous sequences and 0–17 rearrangements were identified (Fig. 3f).

Furthermore, obvious variations in rearrangement rate were identified at both the inter-genus (Fig. 3a, b) and intra-genus (Fig. 3c–f, Additional file 4: Fig. S5) levels. An over 10-fold variation in rearrangement rate (0.16–2.80) occurred among eleven genera, seven of which were lower than one (from 0.16 to 0.84) (Fig. 3b). The estimated common ancestor of Ejap, Msie, Pser, Pbet, and Spoh had a rearrangement rate as low as 0.18 after divergence with *Prunus*, which increased to 0.24 in Ejap, 1.14 in Pser, 2.08 in Msie, 2.45 in Pbet, and 2.80 in Spoh. Within *Malus*, the highest rearrangement rate (7.69 rearrangement events per million years ago (Mya)) was identified in the divergence between *Malus baccata* (Mbac) and the other three species (Additional file 4: Fig. S5a), and the pair-wise rearrangement rates (4.32 to 5.56) between Mbac and the other four samples (Fig 3c) were higher than the others (from 0 to 2.00). In *Pyrus*, 2.88 rearrangement events/Mya were identified in Pcom (Additional file 4: Fig. S5b), and six rearrangement events which occurred at 0.05 Mya resulted in an extremely high rearrangement rate (120 rearrangement events/Mya) which experienced an increase in the pair-wise rearrangement rate between Pysb and Pbre-D (Fig. 3d). Variations in the rearrangement rate were also identified in *Prunus* and *Fragaria* (Fig. 3e, f; Additional file 4: Fig. S5c, d). Nine rearrangement events which occurred about 1.73 Mya in *Prunus avium* (“Glory”: Pavi-G and “Staccato”: Pavi-S) resulted in a higher rearrangement rate than other species in *Prunus* (Additional file 4: Fig. S5c). Two *Fragaria* wild species (*Fragaria mandschurica*: Fman and Fves) experienced a threefold greater increase in rearrangement rate (10.52) than the other wild *Fragaria* species (0–3.35), and the rearrangement rate of *Fragaria ananassa* (“Royal Royce”: Fana-R and Fana-C) was 6.42 (Additional file 4: Fig. S5d).

### Mitogenomes reveal pear maternal phylogeny

**Fig. 4:**
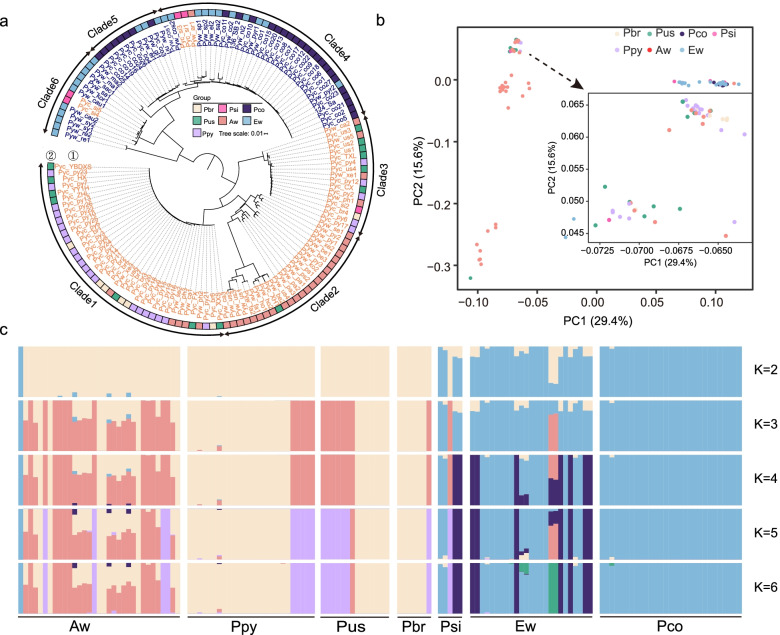
Population structure of 139 domesticated and wild pears. **a** Maximum likelihood phylogenetic tree estimated from SNPs with maf > 0.01 and max missing rate < 0.1. (1) The labels are colored with orange and blue, representing European and Asian pear accessions, respectively. (2) Each pear group is color coded. **b** Principal component analysis (PCA) of the 139 pear accessions. **c** Bayesian model-based clustering of the 139 pear accessions with the number of ancestry kinship (K) ranging from 2 to 6. Each vertical bar represents one pear accession, and the *x*-axis shows the different pear accessions. Each color represents one putative ancestral background, and the *y*-axis quantifies the ancestry membership. Aw, Asian wild pears; Ew, European wild pears; Ppy, *Pyrus pyrifolia*; Pus, *Pyrus ussuriensis*; Pbr, *Pyrus bretschneideri*; Psi, *Pyrus sinkiangensis*; Pco, *Pyrus communis*

The nuclear genome of pears is composed of biparental genetic background due to its self-incompatibility [21]. Compared with the nuclear genome phylogeny, the mitogenome phylogeny reveals the maternal relationship between different pear species. DNA re-sequencing data from 139 pear accessions were mapped to the “Dangshansuli” mitogenome (Additional file 7) to generate a SNP-based matrix, which included 85 Asian (52 cultivated and 33 wild) and 54 European (29 cultivated and 25 wild) pears. Our phylogenetic analysis of the associated mitogenomes revealed two groups, Asian and European pears (Fig. 4a). Among the Asian clade, three subclades were further subdivided, namely clades 1 and 3, which consisted of most of the Asian cultivated pear accessions, while clade 2 contained the wild Asian pear accessions. Cultivars of *Pyrus pyrifolia*, *P. ussuriensis*, and *P. bretschneideri* were mixed in clades 1 and 3. Four *P. sinkiangensis* cultivars clustered in the European group and one in the Asian group. Consistently, PCA (Fig. 4b) and structural analysis (Fig. 4c) also showed that Asian cultivated pears were divided into two groups.

### Identification of selective sweeps and divergent deletion types in mitogenomes

**Fig. 5:**
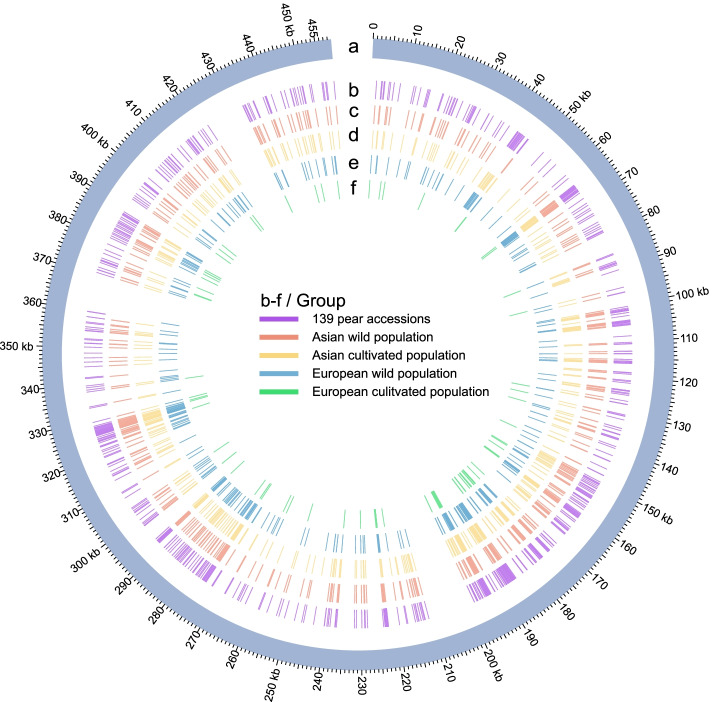
The distribution of SNPs and INDELs across mitogenomes in different groups. **a** The whole mitogenome of “Dangshansuli.” Total variants detected among **b** 139 pear accessions, **c** Asian wild pear population, **d** Asian cultivated pear population, **e** European wild pear population, and **f** European cultivated pear population

**Fig. 6:**
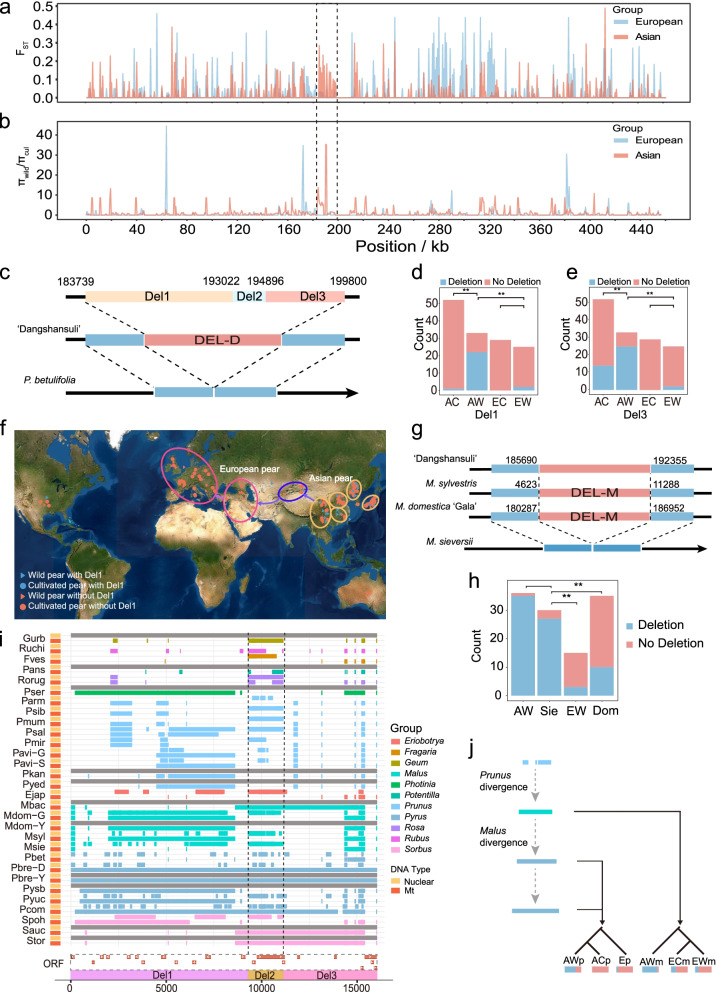
Deletion analysis of the mitogenomes of pear and apple. **a** Distribution of *F*_ST_ values across the whole Asian and European pear populations. **b** Distribution of *π*_wild_/*π*_cul_ ratios; cul means cultivated pear population; dotted regions represent the deletions (Pbre-D: from 183,739 to 199,800 bp) in *P. betulifolia*. **c** A deletion (DEL-D) was identified in *P. betulifolia*. The deletion sequence is further divided into three parts (Del1, Del2, and Del3). **d**, **e** Appearance times of Del1 (**d**) and Del3 (**e**) in the four pear groups. Red represents the samples not containing the deletion, and blue represents the samples containing the deletion. AC, Asian cultivated group; AW, Asian wild group; EC, European cultivated group; EW, European wild group. **f** Geographical distribution of 139 pear accessions. The main distribution areas are marked by circles. Blue indicates pears with the deletion, and red indicates pears without deletion. Triangles represent wild pears, and circles represent cultivated pears. **g** A deletion (DEL-M) is identified in *M. sieversii* which did not appear in *M. sylvestris* and *M. domestica* cv. “Gala.” This deletion is homologous with a sequence (185,690–192,355 bp) of “Dangshansuli.” **h** Appearance times of DEL-M in the four apple groups. Red represents the samples not containing the deletion, and blue represents the samples containing the deletion. Sie, *M. sieversii*; AW, Asian wild group; Dom, cultivated group; EW, European wild group. **i** Homologous sequences of Del1, Del2, and Del3 in 30 mitogenomes and nuclear genomes. The gray bar indicates no information was available. ORFs were identified using ORFfinder with a minimum length of 150 bp. **j** Putative dynamics of evolution and expansion of the deletion sequence during the divergence of Rosaceae species. AWp, Asian wild pear; ACp, Asian cultivated pears; Ep, European pears; AWm, Asian wild apples; ECm, European cultivated apples; EWm, European wild apples. *P*-values were determined by a two-tailed Student’s *t*-test (**P-value < 0.01)

In 139 pear accessions, 1046 SNPs and 118 INDELs were identified (Fig. 5, Table 3), with only 95 SNPs (9.08%, Additional file 8) and two INDELs (1.69%, Additional file 9) being located in genes. To identify the specific regions under selection, selective sweeps were identified based on the diversity of the pear mitogenomes (Fig. 6a, b). For Asian pears, 5.88% (27.00 kb/458.90 kb) of the regions showed selective sweep signatures containing four protein-coding genes and one tRNA (Additional file 10). For European pears, there were selective sweep signatures for 2.18% (10.00 kb/458.90 kb) of sequences, which contained three protein-coding genes. No overlapping selective sweeps were detected between Asian and European pears based on the mitogenomes.

**Table 3:** Summary of the SNPs and INDELs in 139 pear accessions

Population	Total variants	INDEL	SNP
All accessions	1164	118	1046
Asian wild group	815	82	733
Asian cultivated group	806	90	716
European wild group	613	60	553
European cultivated group	194	20	174

One continuous region from 185 to 190 kb showed a selective sweep signature in Asian pears, and *P. betulifolia* had deletions in this region (DEL-D, Pbre-D: 183.74–199.80 kb) (Fig. 6c). DEL-D was divided into three parts (Del1, Del2, and Del3); Del1 and Del3 were mitochondrial-specific sequences, and Del2 was similar to the chloroplast genome sequence (100% BLASTN identity). Therefore, we only analyzed the frequency of Del1 and Del3 in the four pears groups. Sixty-six percent (22/33) of Asian wild pears contained Del1, and the frequency was significantly (chi-square test, *P*-value = 9.39e−15) higher than Asian cultivars (1.92%) (Fig. 6d). However, this divergence did not appear in European pears, and 92% of European wild pears and 100% of European cultivated pears did not contain Del1. This phenomenon also appeared in Del3, for which a significantly different frequency (chi-square test, *P*-value = 3.11e−16) was observed between Asian wild and cultivated pears (Fig. 6e). As pears spread to the Middle East and Europe, most European wild and cultivated pears did not contain the Del1 (Fig. 6f).

A deletion (DEL-M) (*Malus domestica* cv. “Gala”: 180,287–186,952 bp), in a part of Del1, was also identified in *M. sieversii* (Fig. 6g), and DEL-M showed significantly (chi-square test, *P*-value < 0.01) different frequencies between wild and cultivated apples (Fig. 6h, Fig. S6a), based on the re-sequencing data of 116 apple accessions. Eighty percent of apples in the European wild (EW) group and 71.43% in the *M. domestica* (Dom) group did not contain DEL-M. Based on the apple distribution (Additional file 4: Fig. S6b), the *M. sieversii* group was divided into the Sie_X (cultivated in the east of TianShan) and Sie_K (cultivated in the west of Tianshan) groups. One hundred percent of Sie_X and 97.22% of Asian wild (AW) apples contained DEL-M.

Among the Rosaceae mitogenomes, large sequence fragments of DEL-D firstly appeared in Amygdaloideae and then expanded into *Malus* and *Pyrus* (Fig. 6i; Additional file 11). Compared with *Rosoideae*, large fragments (> 1 kb) of Del1 were identified in Amygdaloideae mitogenomes. Lengths of 1589–4201 bp of *Prunus* mitogenomes were mapped to Del1. A total of 8519 bp of sequence in the Pser mitogenome could be mapped to Del1. In *Malus*, a total of 6951 bp of sequence in the Mdom-G, Mdom-Y, and Msyl could be mapped to Del1. Spoh also contained 6133 bp of sequence mapping to Del1. More than 4000 bp of Del3 sequence was identified from the Pbre-D, Pbre-Y, Pcom, Mbac, Sauc, and Stor mitogenomes. Only 681 bp of Del3 was identified from Pysb and Pyuc, and 599–602 bp of mitogenome sequences of *Prunus* was mapped to Del3. Furthermore, the nuclear sequence mapping results showed that less than 10% of DEL-D sequences were shared with the nuclear sequences in *Fragaria*, *Rubus*, and *Rosa*, but this percentage increased in *Prunus* (5.69–44.06%), *Malus* (22.99–55.60%), and *Pyrus* (21.04–33.15%) (Additional file 11).

## Discussion

### Gene loss and genome variation in 38 mitogenomes of Rosaceae

Mitogenomes have variable gene content [27] and genome structure [11]. Thirty-eight mitogenomes from members of the Rosaceae were assembled and annotated to characterize the variations. Consistent with Fabaceae [9] and Poaceae [28], gene loss appeared before Rosaceae speciation, and the loss of *rpl2*, *rps10*, *rps11*, *rps19*, and *rps2* may have occurred in an ancestor of Rosaceae. In addition, *rps2* and *rps11* were lost in all eudicots [27], indicating the more ancient losses of genes *rpl2*, *rps10*, and *rps19*. Within Rosaceae, *rpl16*, *sdh3*, and *rpl5* were absent in five genera of Rosoideae, which represent shrub and herb species, and *rps12* was lost in three herb genera (*Geum*, *Fragaria*, and *Potentilla*) (Fig. 1). These gene losses might affect the translocation and splicing of mitochondrial genes [29] and further influence plant development, reproduction, and other morphological and physiological traits, such as stunting in maize [30], distorted leaves in *Arabidopsis* [31], stress responses in *Oryza sativa* [32], and the parasitic lifestyle of *V. scurruloideum* [4].

In this study, the genome sizes of the 38 mitogenomes were highly correlated with short (< 100 bp) and medium (100 bp ≤ repeat length ≤ 500 bp) repeat lengths (Fig. 2e, f; Additional file 4: Fig. S2a, b, e, f), indicating that repeat sequences may be related to the divergence of mitogenome sizes in Rosaceae. The DNA repair hypothesis suggests that repeat sequences are formed by non-homologous end joining and break-induced replication (BIR) and further drive genome expansion at evolutionary time scales [33]. However, this phenomenon was not consistently observed in 88 seed plant mitogenomes, and several mitogenomes had a burst in repeat sequences, which indicated that other mechanisms may drive genome size variation such as gains or losses of entire chromosomes [34], abundant rearrangements, or loss of non-coding sequences [35].

Although repeats with lengths longer than 1000 bp showed a low correlation with genome size (Additional file 4: Fig. S2) in Rosaceae mitogenomes, they contained higher recombination frequencies than repeats with lengths shorter than 500 bp. Large mitochondrial repeats (> 1000 bp) undergo high-frequency reciprocal recombination to subdivide the genome in other plant species [36]. In addition, twenty repeats had recombination frequencies greater than 50%, indicating that a “master circle” was not the main conformation. High sub-genomic conformations have been observed in vivo, as exemplified in *Silene* [5], *Cucumis* [37], and *Selaginellaceae* [38], and no master conformation appeared in *Saccharum officinarum* [39]. Moreover, more than one repeat containing such recombination frequencies indicated that many conformations may appear at the same time (Additional file 6).

In this study, an over tenfold variation in rearrangement rate occurred between eleven genera of Rosaceae (Fig. 3b), and this variation also occurred within genera (Additional file 4: Fig. S5). In addition, at least 600-fold variation in rearrangement rate was identified in seed plants [8], and some studies found that environmental stress [40–43] and nuclear gene variation (like *MSH1* and *RECA*) [44] might contribute to mitogenome rearrangement. In *Malus*, Mbac originates from Siberia, Msie is distributed in Central Asia, and Msyl is distributed in Western Europe [45]. *Pyrus* spreads from southwest China to Europe [21]. *Fragaria* is widespread in Asia, Europe, and North America [46, 47]. *Prunus* spreads from Asia to Europe [48, 49]. These different geographical distributions and environmental changes might be one reason for the variation in rearrangement rate among Rosaceae species.

### Domestication may have been involved in the evolution and expansion of mitogenomes

Human selection has modified many crop traits, and cultivated crops are divergent from their wild progenitors [50]. DEL-D in selective sweep regions supports that selection drives mitogenome variation in pears. Formed by multi-step processes, DEL-D finally became fixed in Asian cultivated pears during domestication (Fig. 6j). Functional mitochondrial gene formation includes multiple steps and can cause phenotypic changes, biological diversity, and further benefits for natural adaptation [51]. DEL-D was formed by multi-recombination events, sequence imports, and new ORF formations (Fig. 6i), which may become a new resource conferring phenotypic or metabolic changes and contributing to adaptations to environmental stress. Afterwards, selection may quickly drive the allele frequency changes to improve the adaptive ability of the population [52]. DEL-D frequency is very different between Asian cultivated and wild pears and between Asian and European wild pears (Fig. 6d, e). DEL-M also had a significantly different frequency between Asian and European apples and between *M. sieversii* and cultivated apples (Fig. 6h). The selection sweeps and deletion frequency changes might aid in adaptation to environmental changes or be fit for human needs [53, 54].

### Mitochondrial variants shed new insights on the maternal relationships between *Pyrus* species

The topology based on the newly assembled mitogenomes provides insights into the maternal phylogenetic relationships of *Pyrus* species, and it presents an alternative framework to that based on nuclear sequences [21]. Compared with nuclear-based phylogenetic analysis, Asian cultivated pears were divided into clade 1 and clade 3, and three main cultivated pear species (*P. pyrifolia*, *P. bretschneideri*, and *P. ussuriensis*) were mixed in clades 1 and 3, suggesting the mitogenome divergence process produced two main maternal lines in Asian cultivated pears. What is more, the divergence occurred in the maternal parents of *M. domestica*, and the selection of fruit size, flavor, or unilateral compatibility in crosses may be responsible for this divergence [55]. Five *P. sinkiangensis* cultivars were divided into Asian and European groups indicating that the maternal parents of *P. sinkiangensis* came from both Asian and European pears. Most Asian wild pear accessions were divergent from the cultivated species, and *Pyrus calleryana* (Pyw_ca), *Pyrus xerophila* (Pyw_xe), *Pyrus phaeocarpa* (Pyw_ph), and *Pyrus serrulata* (Pyw_se) showed a close relationship with cultivated pears indicating that introgression of maternal parents might happen because of cross-hybridization and adjacent distribution.

## Conclusions

In this study, in-depth comparisons showed the evolutionary patterns of 38 mitogenomes in Rosaceae. Apparent gene losses and shrinkage of the mitogenome size occurred in the Amygdaloideae and Rosoideae subfamilies. Repeat content may lead to genome size variations and primarily drive the dynamics of genome structure by homologous recombination and genomic rearrangements. We estimated the absolute rearrangement rate of Rosaceae mitogenomes, and variations in rearrangement rates were also identified in *Prunus*, *Malus*, *Pyrus*, and *Fragaria* genera. Two divergent maternal lineages were identified in Asian cultivated pears, and free hybridization might explain the mixed maternal lines of cultivated *P. pyrifolia*, *P. ussuriensis*, and *P. bretschneideri*. *Pyrus*-specific sequence variation (DEL-D) was determined, based on the complete mitogenome and population data, to have originated from Amygdaloideae, and this sequence quickly expanded from Asian wild species to Asian cultivated species and European populations. This comparative genomic study provides new insights into the evolutionary and selection patterns of Rosaceae mitogenomes.

## Method and plant materials

### Assembly and annotation of the mitochondrial and chloroplast genomes

Thirty-four of the 38 mitogenomes were assembled using NGS and long-read sequencing data. The Illumina HiSeq 2000 data generated from the whole genome of “Dangshansuli” were used for mitogenome assembly, and the series of the “Dangshansuli” (*P. bretschneideri* Rehd.) published genome BAC libraries were selected (library insertion sizes of 180 bp, 488 bp, 500 bp, 800 bp, 2 kb, 5 kb, 10 kb, and 20 kb) [56]. Fastq files were first filtered using Trimmomatic [57] with default parameters, using 800 bp library insertion size data for mitogenome assembly. Reads were assembled using SOAPdenovo2 [23], and the scaffolds were polished using Pilon v1.23 [58]. Furthermore, to ensure that scaffolds were indeed mitogenomes, we chose the first ten longest assembled scaffolds to do the alignment in the NCBI assembly database, using a cutoff for the BLASTN *e*-value of 1e−5 for the scaffolds [59]. Lastly, scaffolds were selected from the mitogenomes. Insertion sizes of 2 kb, 5 kb, 10 kb, and 20 kb reads helped to concatenate the scaffolds into one, and then the mitogenome was polished by Pilon to fill the gaps. A similar method of organelle genome assembly based on whole genomes has been performed on plants [60].

The raw reads of 33 Rosaceae species were downloaded from NCBI (Additional file 12). We chose the “Dangshansuli,” *Rosa chinensis* (CM009589.1), and *Prunus avium* (MK816392.2) mitogenomes as the reference genomes. The mitochondrial long reads were identified by BLASR [61] to obtain candidate reads from the references and then assembled into contigs using the program Canu v1.8 [24]. Overlaps of mitochondrial candidate contigs were identified using the BLASTN program [59] and finally formed circular molecules. The circular molecules were polished by Pilon v1.23 [58]. Long and short reads were remapped to the polished genome sequences to check the completeness of all newly assembled mitogenomes (Additional file 2). The high-quality complete mitogenomes were annotated by Geseq [62] and Mitofy [63]. The final annotations were checked manually to correct the position of the start and stop codons. A strategy similar to the mitogenome assembly strategy was used for chloroplast genome assembly, and the genome sequences were annotated by Geseq [62]. The annotation files were further checked manually.

### Identification of plastid-derived and repeat sequences

To identify plastid-derived sequences, the 38 mitogenomes were searched against the corresponding plastid genomes in the BLASTN program using an *e*-value cutoff of 1e−6 and a word size of 7, simultaneously. Repeats identified in the 38 mitogenomes were carried out using similar methods [64], and the BLASTN program was used to search each mitogenome against itself, using an *e*-value limit lower than 1e−6 and a word size of 7. The Caper/R package was used to perform the phylogenetic generalized least squares (PGLS) analysis to identify correlations between genome sizes and repeat sequences in the 38 Rosaceae mitogenomes. For the analysis of Fabaceae and seed plants (including 14 and 88 genera), only one accession per genus was chosen.

### Identification of repeat-mediated homologous recombination events

To detect active, repeat-mediated, homologous recombination events within the long sequencing reads, we first built up mitochondrial read databases of 33 mitogenomes (the five other samples were excluded due to lack of long sequencing data). We used the 33 mitogenome assemblies as a reference to obtain candidate mitochondrial sequences from whole DNA long sequencing reads by BLASTN, using an *e*-value cutoff of 1e−100. Candidate mitochondrial reads were further searched against chloroplast genome sequences (Additional file 13) to remove putative plastid reads with overall alignment coverage of > 85% of the read length, and the clean reads were self-corrected using Canu v1.8 [24]. Finally, we obtained 33 mitochondrial read databases (Additional file 14) and used similar methods [13] to identify repeat-mediated homologous recombination events. Briefly, each repeat pair with 200 bp of up- and down-stream sequence was extracted as reference sequences and used to build two recombinant sequences (repeat pairs with 100% BLASTN identity) or six recombinant sequences (repeat pairs were lower than 100% BLASTN identity) (Additional file 4: Fig. S7). Then, the mitochondrial reads were blasted against the reference and recombinant sequences, and reads having identities above 99% and hit coverages of 200 bp in two flanking regions were selected.

### Species tree construction and divergence time estimation

A total of 38 Rosaceae chloroplast genomes (Additional file 13) were used for phylogenetic analysis and divergence time estimation. The coding sequences of 76 chloroplast protein-encoding genes of the 38 Rosaceae samples (Additional file 15) and an outgroup, *Vitis vinifera* (NC_007957), were aligned. Phylogenetic trees were constructed using IQ-TREE [65]. Divergence time estimation was conducted by MCMCtree of PAML 4.9 [66] with the following parameters: burn-in of 5,000,000 iterations, sample frequency of 5000, and the MCMC process was performed 20,000 times. Three calibration points were used: one fossil of *Prunus* found in Shandong (> 44.3 Mya) [67], one fossil of *Rubus* (47.8 to 41.3 Mya) [68], and the estimated divergence time (130 to 123 Mya) between *V*. *vinifera* and Rosaceae [69].

### Rearrangement event identification in Rosaceae mitogenomes

To infer the rearrangement rate between eleven genera, multiple alignments of all pairwise combinations of the mitogenomes of the eleven genera (Pmir, Pser, Gurb, Fves, Ejap, Pbet, Ruchi, Rorug, Pans, Spoh, and Msie) were conducted using Mauve v2.0 [70] to analyze locally collinear blocks (LCBs) in each mitogenome with default parameters, and pairwise rearrangement distances in terms of a minimum number of rearrangements were inferred using GRIMM with the circle chromosome option [71]. To explore the rearrangement rate of different branches of the tree, eleven samples were used in MLGO to infer the ancestral genome arrangement [72]. The rearrangement events between each node and neighboring nodes were calculated by GRIMM [71]. The rearrangement rate was calculated using the rearrangement events by dividing the absolute time of each branch. In addition, the number of pair-wise rearrangements was divided by double divergence time between the two samples to calculate the mean pair-wise rearrangement rate. Pyuc (for *Pyrus*), *Fragaria viridis* (for *Fragaria*), Msyl (for *Malus*), and *Prunus armeniaca* (for *Prunus*) were chosen as the reference genomes for their respective genera to adjust the direction of other mitogenomes for rearrangement analysis, and the rearrangement rate within the genera *Pyrus*, *Malus*, *Prunus*, and *Fragaria* were calculated using the same calculation methods used for inter-genera analysis.

### SNP and INDEL calling of 139 pear accessions

Together, with the published re-sequencing data of 113 pears [21], we also selected another 26 pear accessions to perform next-generation sequencing using the same method on the HiSeq 2000 platform (Additional file 7). We used the “Dangshansuli” mitogenome as a reference for SNP and INDEL calling. Raw data of 139 pear accessions were trimmed by Trimmomatic v0.39 [57]. Clean data was mapped to the reference genome using Burrows-Wheeler Alignment v0.7.16 (BWA) [73]. SAMtools [74] was used to convert the sequence alignment mapping file (SAM) into a binary SAM (BAM) file. Then, the removal of duplicated reads was performed using the Picard software (http://broadinstitute.github.io/picard/). Variant identification and filtering were performed using GATK v4.1.4 [75]. Finally, all SNPs and INDELs with minor allele frequencies (MAF) of > 0.01 and max-missing rate of < 0.1 were extracted for subsequent analysis. SNPeff v4.3t [76] was used for SNP and INDEL annotation.

### Phylogenetic tree construction, PCA, and population structure analysis

All SNPs for each sample were connected one by one as a single locus to make fasta files using an in-house python script, and then IQ-TREE [65] was used to generate the phylogenetic tree with the maximum likelihood method, and the best model was detected using the “MF” function. We set the ultrafast bootstrap replication number as 1000. To evaluate the relationships, PCA and population structure analysis were performed using plink v1.90b [77] and admixture v1.3 [78].

### Diversity analysis and selection sweep identification

Pi (*π*) and *F*_ST_ were calculated by VCFtools v0.1.16 [79] with a 1000-bp sliding window and 500-bp steps in pear. To further identify the regions with signals of selection sweeps in cultivated pears, regions (1000-bp window) with signals for selective sweeps were identified with reference to previous criteria: the top *F*_ST_ > 0.1, *π*_wild_/*π*_cul_ ratio > 2 based on common SNPs in the pear mitogenomes [80].

### Frequency of deletion analysis

To further evaluate the frequency of the deletion (DEL-D) in 139 pear accessions, BEDTools v 2.18 [81] was used to calculate the mapping coverage of DEL-D in the 139 pear accessions. First, DEL-D was divided into three parts (Del1, Del2, and Del3), and the read depths of each part (Idep) were calculated respectively. Furthermore, the whole-genome depth of each accession (Wdep) was calculated. To avoid the differences in sequencing depth in the 139 accessions, we used a ratio of Idep divided by Wdep to evaluate the presence and absence of the deletion. Fortunately, the ratio of Del1 was divided into two levels, namely low (0.24–0.72) and high (6.94–142.98), with a high ratio representing Del1 being present in the mitogenome of this accession and a low ratio representing absence. This phenomenon also appeared in Del3. Due to Del2 sharing homology with chloroplast sequences, we excluded Del2 from further analyses. The frequency of Del1 and Del3 in different pear populations were calculated, and the two-tailed Student’s *t*-test was used to identify the significant differences. The same strategy was used to detect the frequency of the deletion (DEL-M: 6666 bp) in 116 apple accessions.

To detect the origin of the deletion sequence, we used a BLASTN search to detect the homologous sequence in 30 Rosaceae mitochondrial and nuclear genomes. The inferred putative origin of the intracellular transfer and nuclear-shared sequences were identified by performing BLASTN searches of mitogenomes against nuclear DNA, with an *e*-value cutoff lower than 1e−100 and hit length more than 100 bp, and the ggplot2 package (https://cran.r-project.org/web/packages/ggplot2/index.html) was used for visualization. An in-house python script was used to calculate the total length of homologous sequences from each mitochondrial and nuclear genome. ORFs with a minimum length of 150 bp were identified in DEL-D using ORFfinder (https://www.ncbi.nlm.nih.gov/orffinder/).

## Supplementary Information

**Additional file 1.** Released Rosaceae mitogenomes (Last access date: 20-Jan-2022).

**Additional file 2.** Mapping depth of 34 newly assembled Rosaceae mitogenomes.

**Additional file 3.** Repeat statistics of 38 Rosaceae mitogenomes.

**Additional file 4: Figure S1.** The relationship between genome size and total repeat length and number in 38 Rosaceae mitogenomes. The repeats were divided into four types: length <100 bp (a, e); 100 bp ≤ repeat length ≤ 500 bp (b, f); 500 bp < repeat length ≤ 1,000bp (c, g); and repeat length >1,000 bp (d, h). The linear regression equation is displayed with adjusted R-square and *P*-values. **Figure S2.** The relationship between mitogenome size and total repeat length and count in 14 Fabaceae mitogenomes. The repeats were divided into six types: all repeats (a, g); repeat length <100 bp (b, h); 100 bp ≤ repeat length ≤ 500 bp (c, i); 500 < repeat length ≤ 1,000 bp (d, j); length >1,000 bp (e, k); and repeat length ≤500 bp (f, l). The linear regression equation is displayed with adjusted R-square and *P*-value. **Figure S3.** The relationship between mitogenome size and total repeat length and count in 88 seed plants. The repeats were divided into six types: all repeats (a, g); repeat length <100 bp (b, h); 100 bp ≤ repeat length ≤ 500 bp (c, i); 500 < repeat length ≤ 1,000 bp (d, j); repeat length >1,000 bp (e, k); and repeat length ≤500 bp (f, l). The linear regression equation is displayed with adjusted R-square and *P*-value. **Figure S4.** The distribution of repeat count (a) and total repeat length (b) of 50 seed plant mitogenomes with genome sizes ranging from 271.60 to 525.67 kb. **Figure S5.** The rearrangement rate estimated using tree-based methods in *Malus* (a), *Pyrus* (b), *Prunus* (c) and *Fragaria* (d). Red numbers on the branches represent rearrangement events and rates (rearrangement events per million years), respectively. Yellow triangles represent the varieties within specie, and the rearrangement events and rates are calculated between species and neighboring nodes. The blue bar indicates the 95% highest posterior densities. **Figure S6.** The mapping depth and distribution analysis of 116 apple accessions. (a) The mapping depth of 116 apple accessions. The NGS reads are mapped to the *Malus domestica* cv. ‘Gala’ (Mdom-G) mitogenome. A ratio of Idep divided by Wdep was used to evaluate the mapping results, and the ratio was further normalized using the z-score method. Orange: high mt read mapping depth, blue: low mt read mapping depth. AW: Asian wild apples; EW: European wild apples; Sie: *Malus sieversii*; Dom: *Malus domestica*. (b) Distribution analysis of apple mitogenomes. Main distribution areas are marked by circles. Blue: apples containing the deletion (Del), red: apple not containing this deletion. Triangles represent wild apple and circles represents cultivated apple. Dom: *Malus domestica*; Syl: *Malus sylvestris*; Sie_K: *Malus sieversii* in west of TianShan; Sie_X: *Malus sieversii* in east of TianShan; Bac: *Malus baccata*; Asi: *Malus Asiatica*; Hup: *Malus hupehensis*. **Figure S7.** Flow chart for repeat recombination analysis. (a) Recombinant sequence construction. ‘b’ and ‘e’ indicate repeat sequences; ‘a’ and ‘d’ indicate the upstream 200 bp sequences; ‘c’ and ‘f’ indicate the downstream 200 bp sequences. (b) Mitochondrial reads mapping to reference and recombinant sequences using BLASTN. (c) Recombination frequency calculation.

**Additional file 5.** Repeat statistics of 88 seed plant mitogenomes.

**Additional file 6.** Information on 341 repeats containing recombination activities.

**Additional file 7.** 139 wild and cultivated pear accessions and mapping profile.

**Additional file 8.** SNPs annotation of mitogenome.

**Additional file 9.** INDELs annotation of mitogenome.

**Additional file 10.** Mitochondrial genes in selective sweep regions.

**Additional file 11.** Homologous sequence of DEL-D (Pbre-D: 183,739-199,800 bp) in 30 mitogenomes and nuclear genomes.

**Additional file 12.** Project information for the raw sequences of 34 Rosaceae samples.

**Additional file 13.** Summary of 38 Rosaceae chloroplast genomes.

**Additional file 14.** Information for the mitochondrial read databases.

**Additional file 15.** Genes used in the chloroplast genome phylogeny analysis.

## Data Availability

Raw WGS data of pear and apple accessions were downloaded from the NCBI BioProject (PRJNA381668, PRJNA675194, PRJNA844501, and PRJNA322175). The NGS and Pacbio data used for mitogenome assembly were downloaded from NCBI, and the BioProject ID was supplied in Additional file 12. The 34 new assembly mitogenome sequences were all submitted to the NCBI database, and accession numbers are listed in Table 1.
