# Supplementary material for: Rearrangement and domestication as drivers of Rosaceae mitogenome plasticity
Source: BMC Biol. 2022 Aug 19;20:181. doi: 10.1186/s12915-022-01383-3 (PMC9392253; doi:10.1186/s12915-022-01383-3)
Supplement: Supplementary file 4 — Additional file 4: Figure S1. The relationship between genome size and total repeat length and number in 38 Rosaceae mitogenomes. The repeats were divided into four types: length <100 bp (a, e); 100 bp ≤ repeat length ≤ 500 bp (b, f); 500 bp < repeat length ≤ 1,000bp (c, g); and repeat length >1,000 bp (d, h). The linear regression equation is displayed with adjusted R-square and P-values. Figure S2. The relationship between mitogenome size and total repeat length and count in 14 Fabaceae mitogenomes. The repeats were divided into six types: all repeats (a, g); repeat length <100 bp (b, h); 100 bp ≤ repeat length ≤ 500 bp (c, i); 500 < repeat length ≤ 1,000 bp (d, j); length >1,000 bp (e, k); and repeat length ≤500 bp (f, l). The linear regression equation is displayed with adjusted R-square and P-value. Figure S3. The relationship between mitogenome size and total repeat length and count in 88 seed plants. The repeats were divided into six types: all repeats (a, g); repeat length <100 bp (b, h); 100 bp ≤ repeat length ≤ 500 bp (c, i); 500 < repeat length ≤ 1,000 bp (d, j); repeat length >1,000 bp (e, k); and repeat length ≤500 bp (f, l). The linear regression equation is displayed with adjusted R-square and P-value. Figure S4. The distribution of repeat count (a) and total repeat length (b) of 50 seed plant mitogenomes with genome sizes ranging from 271.60 to 525.67 kb. Figure S5. The rearrangement rate estimated using tree-based methods in Malus (a), Pyrus (b), Prunus (c) and Fragaria (d). Red numbers on the branches represent rearrangement events and rates (rearrangement events per million years), respectively. Yellow triangles represent the varieties within specie, and the rearrangement events and rates are calculated between species and neighboring nodes. The blue bar indicates the 95% highest posterior densities. Figure S6. The mapping depth and distribution analysis of 116 apple accessions. (a) The mapping depth of 116 apple accessions. The NGS reads are mapp [file 12915_2022_1383_MOESM4_ESM.docx]

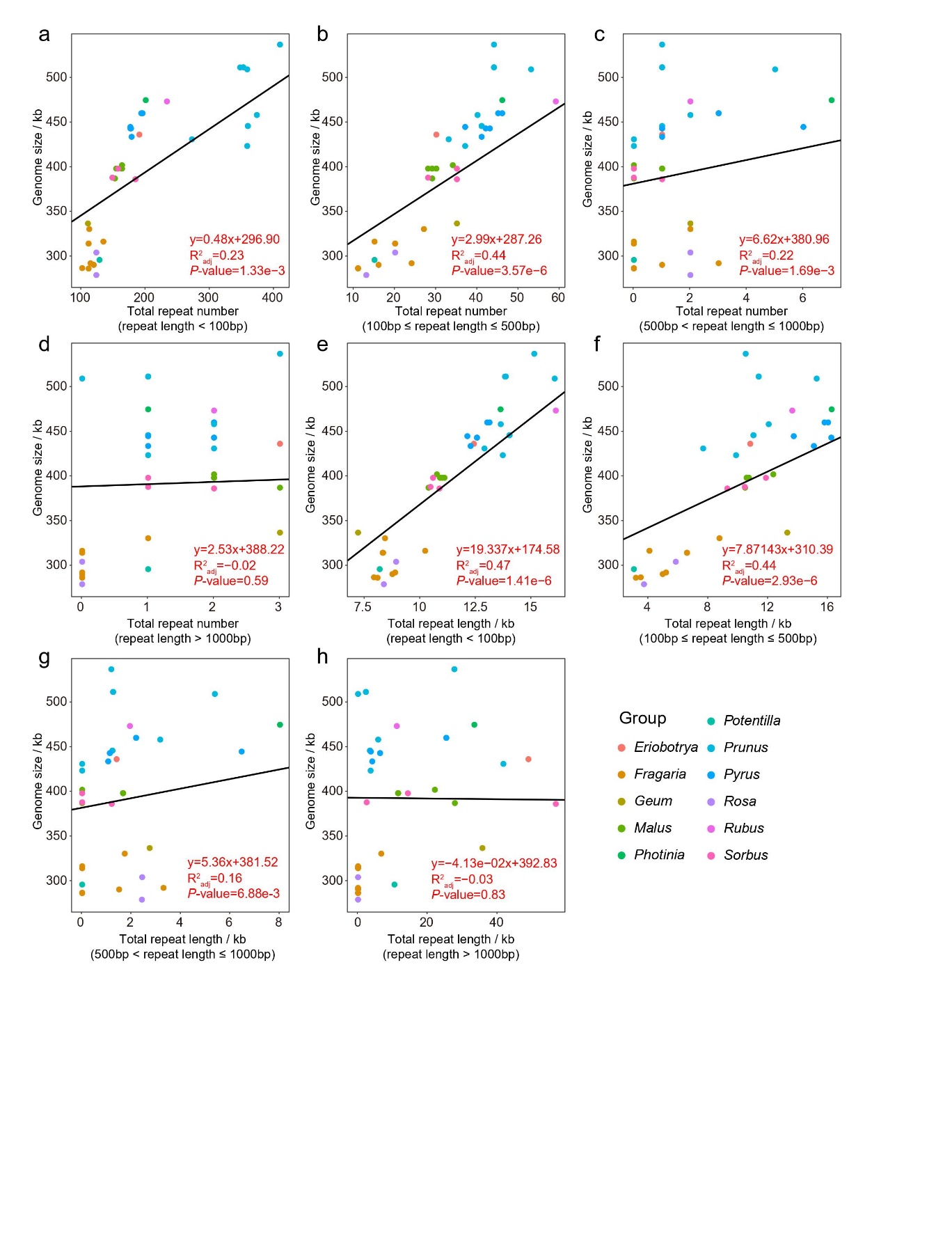


**Figure S1: The relationship between genome size and total repeat length and number in 38 Rosaceae mitogenomes.** The repeats were divided into four types: length <100 bp (a, e); 100 bp ≤ repeat length ≤ 500 bp (b, f); 500 bp < repeat length ≤ 1,000bp (c, g); and repeat length >1,000 bp (d, h). The linear regression equation is displayed with adjusted R-square and *P*-values.


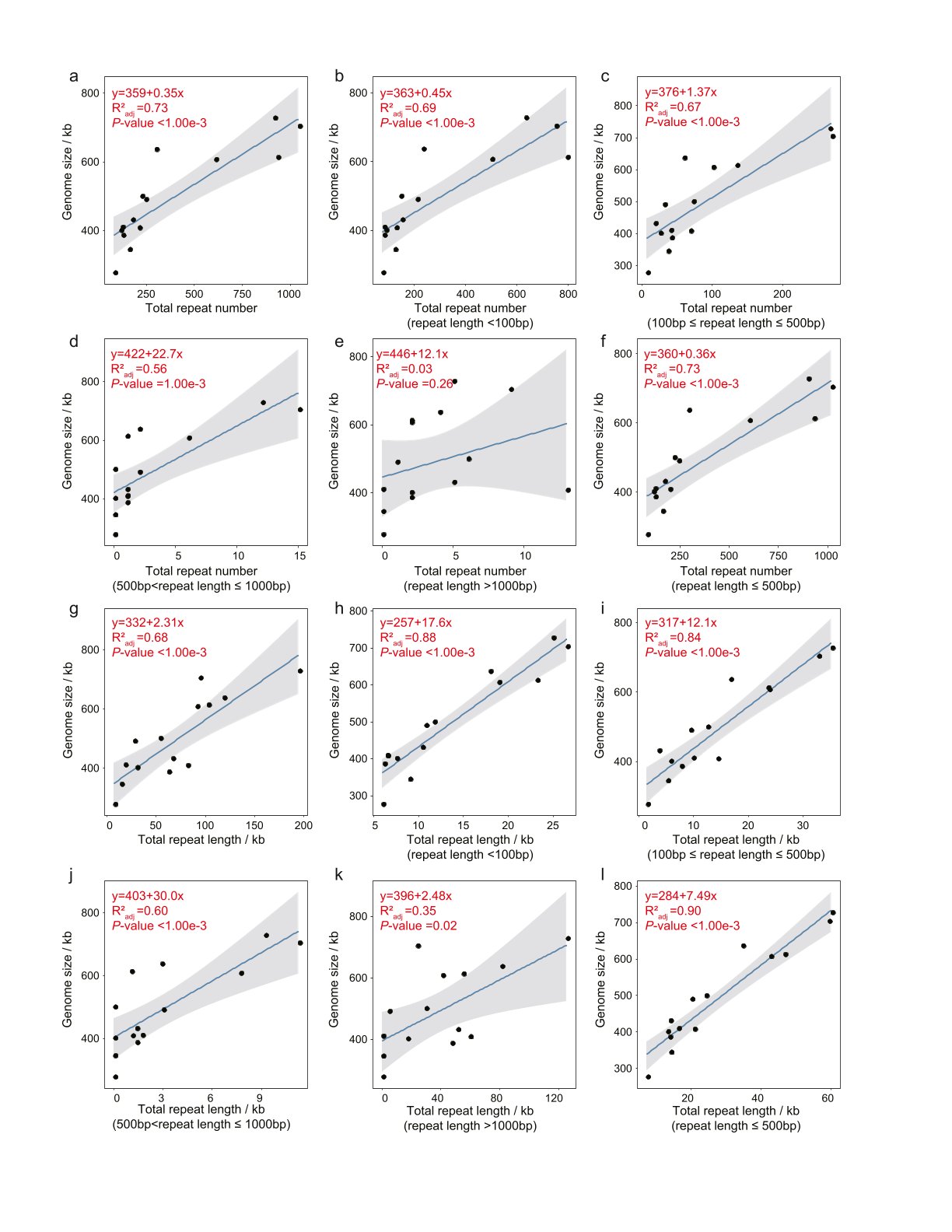


**Figure S2: The relationship between mitogenome size and total repeat length and count in 14 Fabaceae mitogenomes.** The repeats were divided into six types: all repeats (a, g); repeat length <100 bp (b, h); 100 bp ≤ repeat length ≤ 500 bp (c, i); 500 < repeat length ≤ 1,000 bp (d, j); length >1,000 bp (e, k); and repeat length ≤500 bp (f, l). The linear regression equation is displayed with adjusted R-square and *P*-value.

**
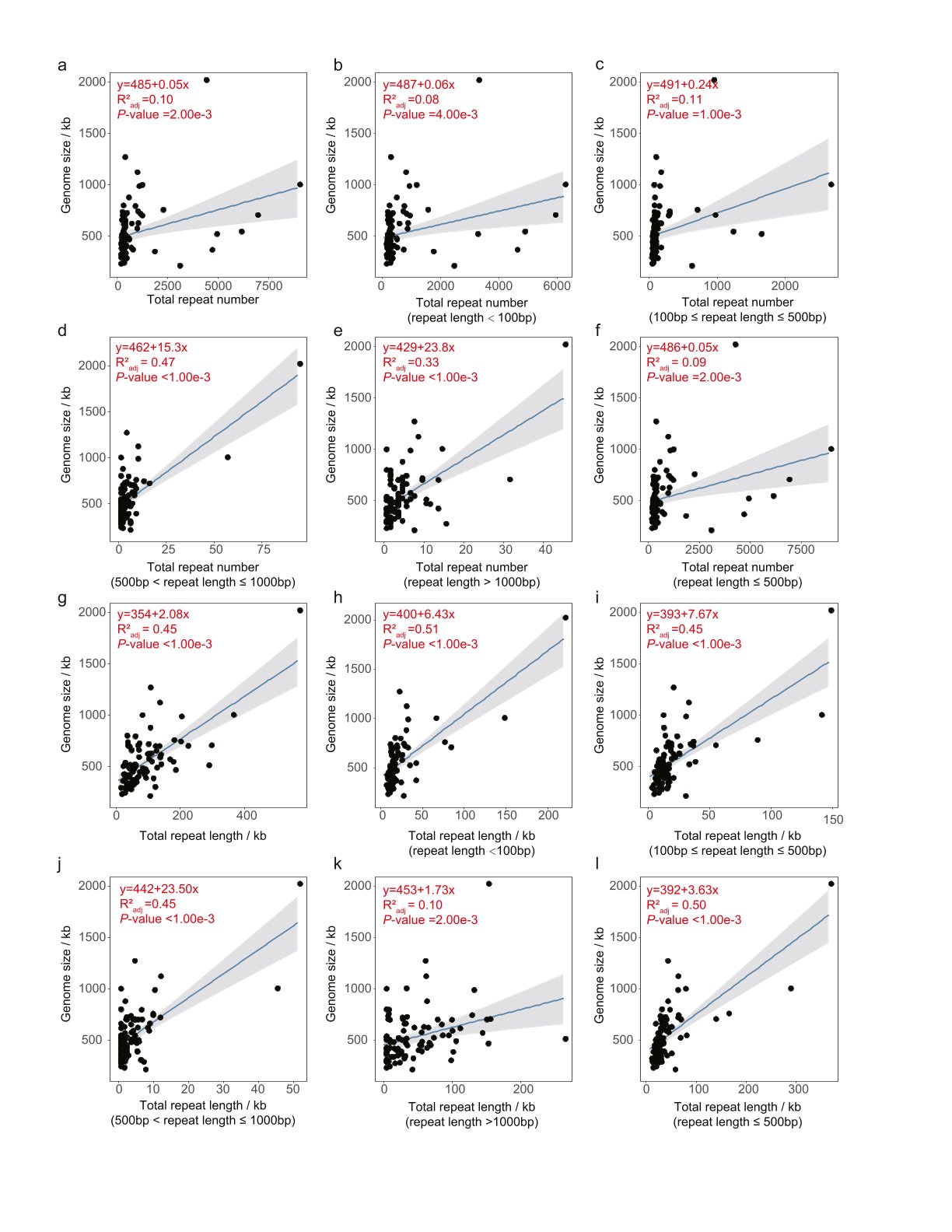
**

**Figure S3: The relationship between mitogenome size and total repeat length and count in 88 seed plants.** The repeats were divided into six types: all repeats (a, g); repeat length <100 bp (b, h); 100 bp ≤ repeat length ≤ 500 bp (c, i); 500 < repeat length ≤ 1,000 bp (d, j); repeat length >1,000 bp (e, k); and repeat length ≤500 bp (f, l). The linear regression equation is displayed with adjusted R-square and *P*-value.


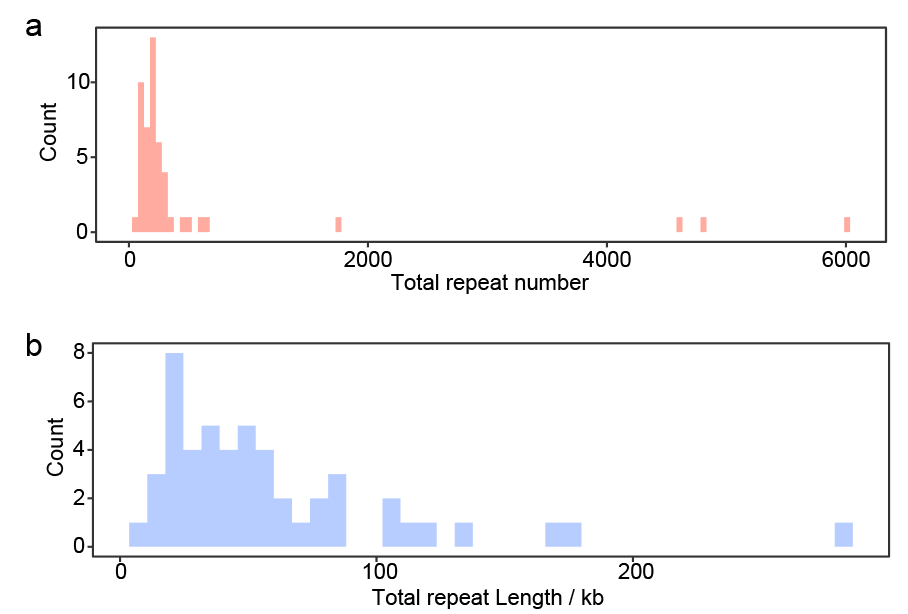


**Figure S4: The distribution of repeat count (a) and total repeat length (b) of 50 seed plant mitogenomes with genome sizes ranging from 271.60 to 525.67 kb.**


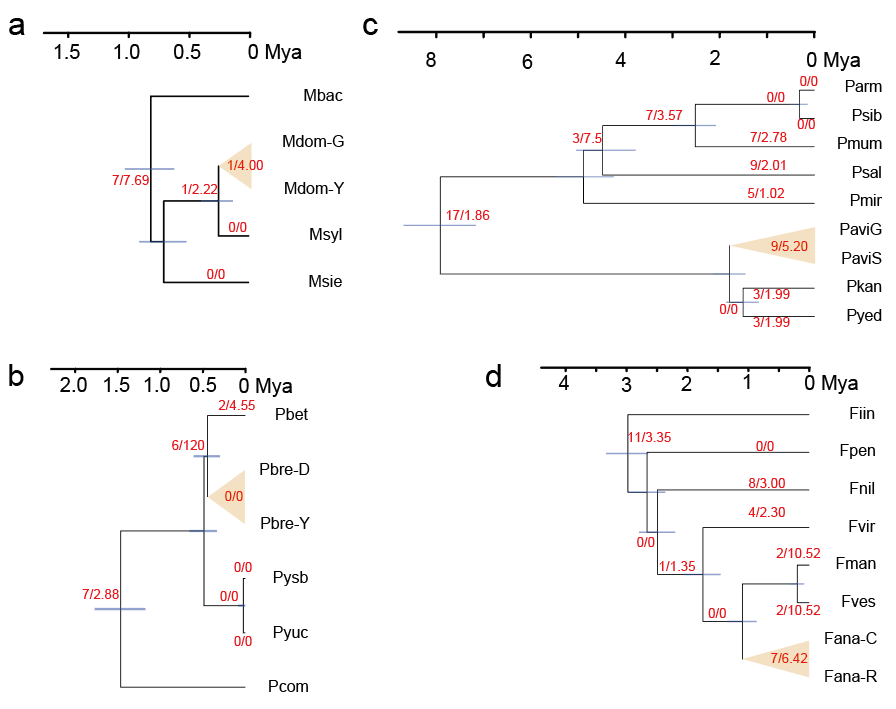


**Figure S5: The rearrangement rate estimated using tree-based methods in *Malus* (a), *Pyrus* (b), *Prunus* (c) and *Fragaria* (d).** Red numbers on the branches represent rearrangement events and rates (rearrangement events per million years), respectively. Yellow triangles represent the varieties within specie, and the rearrangement events and rates are calculated between species and neighboring nodes. The blue bar indicates the 95% highest posterior densities.


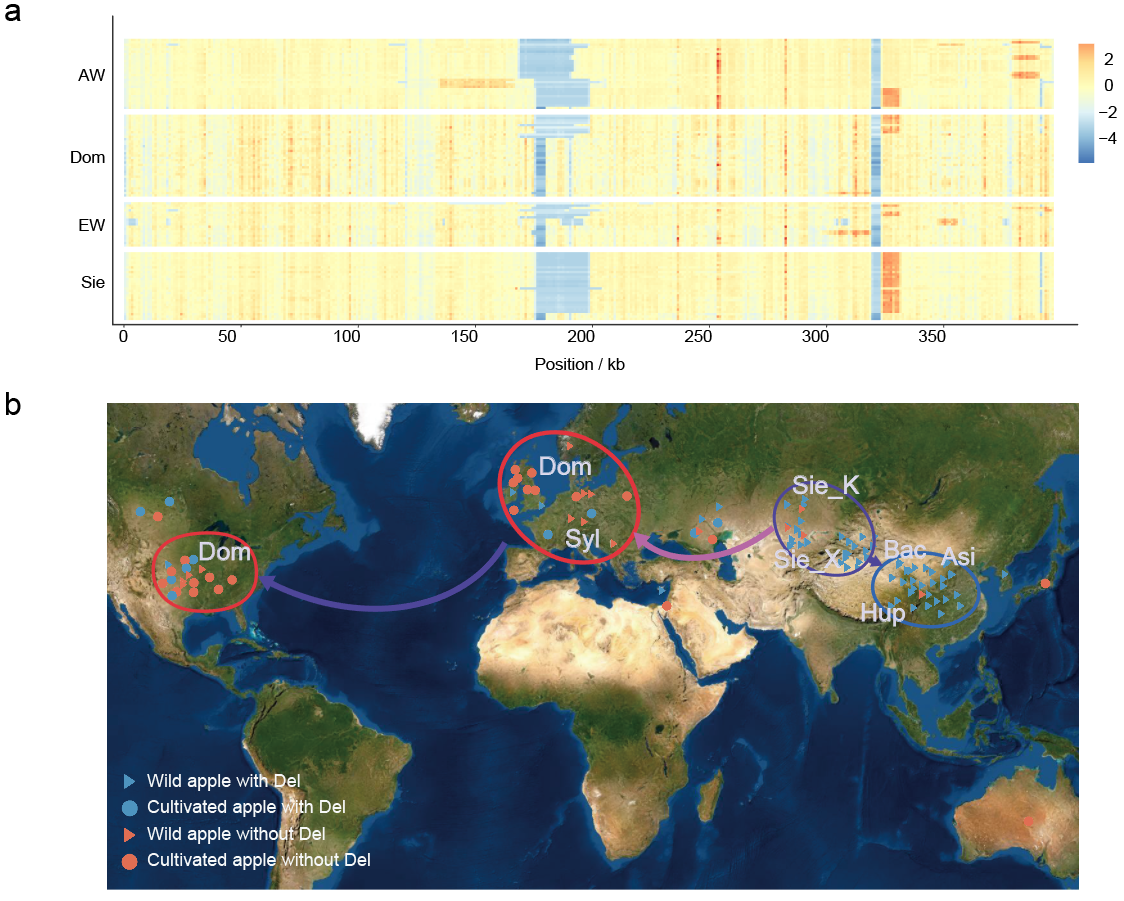


**Figure S6: The mapping depth and distribution analysis of 116 apple accessions.** (a) The mapping depth of 116 apple accessions. The NGS reads are mapped to the *Malus domestica* cv. ‘Gala’ (Mdom-G) mitogenome. A ratio of Idep divided by Wdep was used to evaluate the mapping results, and the ratio was further normalized using the z-score method. Orange: high mt read mapping depth, blue: low mt read mapping depth. AW: Asian wild apples; EW: European wild apples; Sie: *Malus sieversii*; Dom: *Malus domestica*. (b) Distribution analysis of apple mitogenomes. Main distribution areas are marked by circles. Blue: apples containing the deletion (Del), red: apple not containing this deletion. Triangles represent wild apple and circles represents cultivated apple. Dom: *Malus domestica*; Syl: *Malus sylvestris*; Sie_K: *Malus sieversii* in west of TianShan; Sie_X: *Malus sieversii* in east of TianShan; Bac: *Malus baccata*; Asi: *Malus Asiatica*; Hup: *Malus hupehensis*.


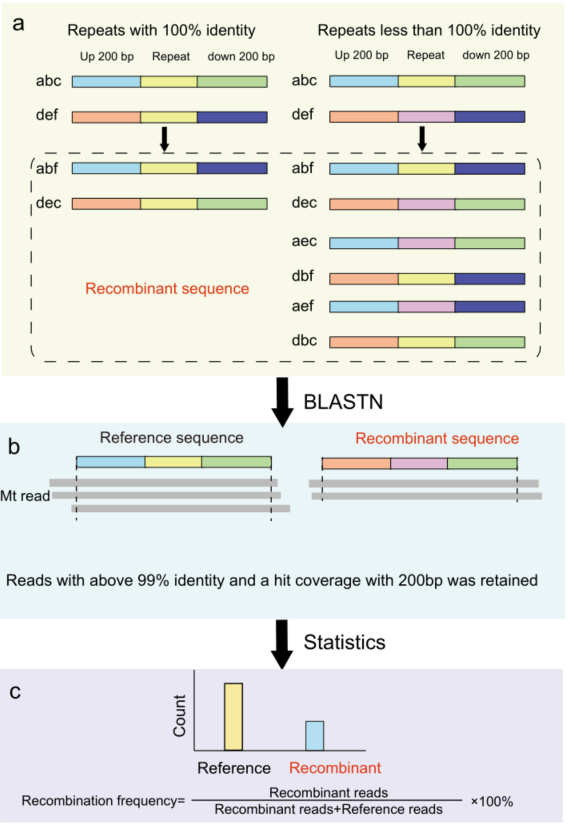


**Figure S7:** **Flow chart for repeat recombination analysis.** (a) Recombinant sequence construction. ‘b’ and ‘e’ indicate repeat sequences; ‘a’ and ‘d’ indicate the upstream 200 bp sequences; ‘c’ and ‘f’ indicate the downstream 200 bp sequences. (b) Mitochondrial reads mapping to reference and recombinant sequences using BLASTN. (c) Recombination frequency calculation.
